# Integrated analysis reveals important differences in the gut and oropharyngeal microbiota between children with mild and severe hand, foot, and mouth disease

**DOI:** 10.1080/22221751.2023.2192819

**Published:** 2023-03-31

**Authors:** Nan Zhang, Danlei Mou, Tongzeng Li, Zhiyun Chen, Chunhua Ma, Lianchun Liang, Qiushui He

**Affiliations:** a Department of Medical Microbiology, Capital Medical University, Beijing, People’s Republic of China; b Department of Infectious Disease, Beijing Youan Hospital, Capital Medical University, Beijing, People’s Republic of China; c Institute of Biomedicine, University of Turku, Turku, Finland

**Keywords:** Gut microbiota, oropharyngeal microbiota, hand, foot and mouth disease (HFMD), 16S rRNA sequencing

## Abstract

Little is known about alternation and difference in gut microbiota between patients with mild and severe hand, foot, and mouth disease (HFMD). We investigated the differences in gut and oropharynx microbiota between mild and severe HFMD in young children and changes in bacterial profiles as the disease progresses from acute to convalescent phase. Forty-two patients with confirmed HFMD were studied, among which 32 had severe HFMD and 10 had mild HFMD. First rectal swabs were collected from all patients at an average of 2 days (acute phase) after the onset of symptoms, and second rectal swabs were collected from 8 severe patients at day 9 (convalescent phase) after the onset. Oropharyngeal swabs were obtained from 10 patients in the acute phase and 6 in the convalescent phase. 16S rRNA sequencing was performed for all 70 samples. Compared with mild HFMD, severe HFMD exhibited significantly decreased diversity and richness of gut microbiota. Gut microbiota bacterial profiles observed in the acute and convalescent phases resembled each other but differed from those in mild cases. Additionally, 50% of patients with severe HFMD in the acute phase harboured a dominant pathobiontic bacterial genus. However, none of the patients with mild HFMD had such bacteria. Similar bacterial compositions in oropharynx microbiota were detected between mild and severe cases. Our findings indicate that severe HFMD exhibits significantly impaired diversity of gut microbiota and frequent gut and oropharyngeal inflammation-inducing bacteria. However, the results should be interpreted with caution as the number of subjects was limited.

## Introduction

Hand, foot, and mouth disease (HFMD) is a common and contagious gastrointestinal disease in infants and children less than 5 years old in Asian countries [1]. In China, it is one of the top three Class C notifiable infectious diseases [2]. HFMD is usually a mild and self-limiting disease. However, severe complications of the nervous and respiratory system, such as encephalitis and pulmonary hemorrhage, can occur, which often lead to death or permanent paralysis [3]. Human enteroviruses A71 (EV-A71) and coxsackievirus A16 (CV-A16) are common viruses responsible for outbreaks of HFMD [4]. During these outbreaks, high morbidity and mortality of severe cases have been reported [5,6].

A study showed that the composition of the gut microbiota of patients with HFMD differed from that of healthy controls [7]. In patients with HFMD, relative abundances (RAs) of *Prevotella* and *Streptococcus* were higher, whereas *Bifidobacterium* and *Faecalibacterium* were depleted [8]. Li et al. recently reported that a reduction in bacterial diversity of gut microbiota was observed in children with HFMD. Particularly, a reduction in two butyrate-producing bacteria, namely *Ruminococcus* and *Roseburia*, and increased number of inflammation-inducing bacteria, namely *Escherichia* and *Enterococcus,* were observed [9]. Moreover, studies on the oral microbiome have revealed that an elevated level of *Streptococcus spp.* is the most important signature of patients with symptomatic HFMD, which shows a positive correlation with the level of enterovirus A RNA [10]. Therefore, both gut and oropharyngeal microbiota may play an important role in pathogenesis and host inflammatory responses in HFMD.

The exact pathogenic mechanisms of severe HFMD remain unclear. Viruses are speculated to target the gastrointestinal epithelium in humans and utilize intestinal bacteria to enhance their own multiplication, pathogenesis, and transmission, thereby leading to the development of HFMD [11]. Gut microbiota work in conjunction with the intestinal barrier to orchestrate a defense network that impedes the invasion of pathogens and maintains gut homeostasis and functionality [12,13]. Therefore, dysbiosis of the gut microbiota could promote viral infection through intestinal barrier disruption by influencing self-renewal of epithelial cells, secretion of the mucus layer, and tight junctions of intestinal epithelial cells, thereby regulating the host immune response [14–17].

Recently, Qin et al. reported that patients with severe influenza showed significant differences in the oropharyngeal microbiota with a super-dominant pathobiontic bacterial genus (SDPG) compared with patients with mild influenza [18]. These SDPGs included *Lactococcus*, *Acinetobacter*, *Streptococcus*, *Corynebacterium*, *Staphylococcus*, and *Prevotella*, and were associated with secondary bacterial infection and death. Moreover, the same group of researchers detected SDPGs in the upper and lower respiratory tract samples of patients with severe coronavirus disease 2019 (COVID-19) [19].

Previous studies have mainly focused on the differences in gut microbiota between patients with HFMD and healthy controls. In Chinese healthy children, *Streptococcus* (RA: 28.5–31.8%), *Prevotella* (RA: 16.1–17.3%), *Neisseria* (RA: 12.6–13.1%), *Veillonella* (RA: 8.1–9.0%), and *Haemophilus* (RA: 6.2–6.3%) have been shown to be the dominant bacterial genera in the oropharynx [20,21]. Meanwhile, the dominant bacterial genera in the gut were *Bacteroides* (RA: 28.0–33.9%), *Faecalibacterium* (RA: 9.2–12.5%), *Prevotella* (RA: 4.4–5.1%), and *Bifidobacterium* (RA: 3.0–3.1%) [22,23]. However, little is known about the dynamic changes in gut and oropharyngeal microbiota during the onset of the disease.

In this study, we aimed to investigate whether there were differences in gut and oropharyngeal microbiota between patients with mild HFMD and patients with severe HFMD, as well as changes in the microbiota profiles from acute to convalescent phase of severe HFMD. Additionally, we explored the possible relations between oropharyngeal microbiota and gut microbiota.

## Materials and methods

### Study participants

This study was approved by the Ethics Committee of Beijing Youan Hospital, Capital Medical University, Beijing, China (No. 15JL13). Written informed consent was obtained from the parents of each pediatric patient. Altogether, 42 patients were included during May 2015–October 2016. All patients resided in northern China. Among the patients, 10 exhibited mild symptoms and 32 were hospitalized and exhibited severe symptoms. For patients with severe symptoms, oropharyngeal swabs and rectal swabs were collected in the acute and convalescent phase, respectively. The median number of days between the two longitudinal swabs was 7 days (range: 5–12 days). The laboratory diagnostic methods were performed in accordance with the approved guidelines [24].

Clinical diagnosis was performed according to the Chinese guidelines for the diagnosis and treatment of HFMD issued by the Chinese Ministry of Health [24]. Patients with more than one of the following HFMD complications were classified as severe case: encephalitis, aseptic meningitis, acute flaccid paralysis, myoclonic jerk, limb weakness, limb jitters and/or astasia, and central cardiopulmonary complications, including pulmonary oedema, pulmonary hemorrhage, circulatory failure, or other critical conditions [24]. The remaining patients with HFMD who visited the outpatient clinic and did not meet the criteria for severe cases, were enrolled as mild cases. The exclusion criteria for all participants included: patients suffered from HFMD but presented other respiratory infection symptoms, digestive tract disease, and patients receiving antibiotic therapy and immunosuppressive drugs within the last 4 weeks. Infants with mothers who had severe obstetric complications, such as gestational diabetes, pregnancy hypertension, preeclampsia, or eclampsia, were excluded. Demographic and clinical information regarding age, gender, fever, consciousness, cough, diarrhoea, treatment, and outcome were collected from the electronic medical records. In addition, serious neurological complications, including encephalopathy, aseptic meningitis, encephalitis, encephalomyelitis, and poliomyelitis-like paralysis, and other severe complications, including myocardial damage, heart failure, neurogenic pulmonary oedema, severe pneumonia, and respiratory failure data, were also collected.

### Clinical stage of patients with HFMD

HFMD cases were classified into five distinct stages according to the Chinese guidelines for the diagnosis and treatment of HFMD based on clinical severity [24]. In this study, cases of stages II to V were categorized into severe ones. Furthermore, stages II, III, and IV referred to the acute phase. Stage V referred to patients whose CNS and cardiopulmonary functions gradually recovered and were thus considered as the convalescent phase. Samples for the convalescent phase were collected on the day of discharge of patients when their condition reached the clinical cure standard.

### Sample collection

Of the 10 patients with mild symptoms, 4 oropharyngeal swabs and 10 rectal swabs were collected. Of the 32 patients with severe symptoms, 32 rectal swabs (1 per patient) and 10 oropharyngeal swabs were collected in the acute phase, and 8 rectal swabs and 6 oropharyngeal swabs were collected in the convalescent phase. Both oropharyngeal and rectal swabs were collected for acute and convalescent phases for six patients (B1, B11, B12, B16, B20, and B31). Both oropharyngeal and rectal swabs were collected for four patients (B2, B23, B24, and B33) at acute phase. Rectal swabs were collected for two patients (B44 and B47) at both acute and convalescent phases. Only rectal swabs were collected at acute phase for the remaining 20 patients with severe symptoms. Samples were collected using a swab containing bacterial DNA Locker (Youkang, Nanjing, China) and subsequently transported within 30 min from the hospital to the laboratory in an ice bag using insulating polystyrene foam containers. In the laboratory, the swabs were immediately stored at −80°C and were thawed immediately prior to DNA extraction.

### Aetiological investigation

Oropharyngeal and rectal swabs were collected from patients with suspected HFMD to specifically detect EV-A71 and/or CV-A16 and other general enteroviruses. Viral RNA was extracted using a Viral RNA Mini Extraction kit (#52904; Qiagen, Hilden, Germany). Then, OneStep RT–PCR kit (#210212; Qiagen) was used for transcription and amplification. Sets of RT–PCR primers used were as follows for EV-A71: forward primer 5′-GCAGCCCAAAAGAACTTCAC-3′, reverse primer 5′-ATTTCAGCAGCTTGGAGTGC-3′; for CV-A16: forward primer 5′-ATTGGTGCTCCCACTACAGC-3′, reverse primer 5′-TCAGTGTTGGCAGCTGTAGG-3′; and for other general enteroviruses: forward primer 5′-TCCGGCCCCTGAATGCGGCTAATCC-3′, reverse primer 5′-ACACGGACACCCAAAGTAGTCGGTCC-3′.

### Genomic DNA extraction and 16S rRNA gene sequencing

Bacterial genomic DNA was extracted using the QIAamp Fast DNA Stool Mini kit (Qiagen) according to the manufacturer's instructions. The V4–V5 regions of the prokaryotic 16S rRNA gene were amplified using the universal primer pair 515F (5′-GTGYCAGCMGCCGCGGTA-3′) and 909R (5′-CCCCGYCAATTCMTTTRAGT-3′) with barcode, sequenced, and then analysed. The V4–V5 regions were chosen because we also wanted to detect low-abundance bacteria [25]. The barcoded amplicons from all samples were normalized, pooled to construct the sequencing library, and then sequenced using MiSeq (Illumina, USA) to generate pair-ended reads with 250 nt length.

The paired-end reads from the DNA fragments were merged using FLASH v1.2.7 [26]. Sequencing data were analysed using Quantitative Insights into Microbial Ecology v1.9.1 and R software v4.1.3 [27]. Operational taxonomic units (OTUs) were clustered with 97% sequence similarity using UPARSE v7.0.1001. The normalized OTU tables were used for diversity and statistical analyses. Bacterial diversity of the samples (alpha diversity) was calculated with observed species, Chao 1, Abundance-based Coverage Estimator (ACE), Shannon index, and Simpson index [28]. Structure of microbial communities (beta-diversity) was calculated using weighted UniFrac distances [29]. Curtis similarity clustering analysis was used to perform principal coordinate analysis (PCoA). Function prediction analysis was performed using PICRUSt [30].

### Statistical analysis

Data were analysed using IBM SPSS statistics v24.0 software for Windows (IBM Corp., Armonk, NY, USA), GraphPad Prism v6.0 software (GraphPad, La Jolla, CA, USA), and Image R software v4.1.3 (Boston, MA, USA). The patients’ ages and the onset of disease when swab samples were taken are expressed as medians and interquartile ranges (IQRs). The Wilcoxon rank-sum test was used to compare the Observed species, Chao1, ACE, Shannon index, and Simpson index between groups. The linear discriminant analysis (LDA) effect size (LEfSe) model was used to identify the differences in microbiota composition for phylotypes [31]. Based on the normalized RA matrix, taxa with significantly different abundances were determined by LEfSe using Kruskal–Wallis rank-sum test. For comparison between groups, *t*-test was performed. Rates or percentages were calculated using the *χ*^2^ test or the Fisher's Exact test. A two-sided *p* < 0.05 indicated a significant difference. The threshold logarithmic LDA score for discriminative features was set as 3.

## Results

### Demographic data and results of the aetiological investigation

The age of patients ranged from 12 to 36 months and the median age was 26 months (Table 1). Male and female sex ratio was 22:20. Of the 32 patients with severe HFMD, 17 were demonstrated to be EV-A71-positive, 3 were CV-A16-positive, and 10 were positive for general enteroviruses. Of the 10 patients with mild HFMD, only 1 was CV-A16-positive. All patients with severe HFMD were hospitalized and were eventually discharged when their condition reached the clinical cure standard.

**Table 1.** Demographic characteristics of patients with hand, foot, and mouth disease (HFMD) included in the study.

	Patients with mild HFMD (*n* = 10)	Patients with severe HFMD at acute phase (*n* = 32)	Patients with severe HFMD at convalescent phase (*n* = 8)
Age (months)^a^	30 (13–36)	26 (14–37)	24 (14–39)
Male (%)	6 (60)	16 (50)	4 (50)
Onset of disease when swab samples were taken (days)^b^	1 (0–2)	2 (0–3)	9 (7–12)
No. of oropharyngeal swabs	4	10	6
No. of rectal swabs	10	32	8

^a,b^ Indicated as median (interquartile range).

### Gut bacterial profiles in patients with severe and mild HFMD

A total of 3,904,495 tags were generated and clustered into 14,438 OTUs. Correspondingly, a total of 37 phyla, 64 classes, 97 orders, 152 families, 330 genera, and 259 species were annotated from the rectal swabs of all patients.

**Figure 1.**
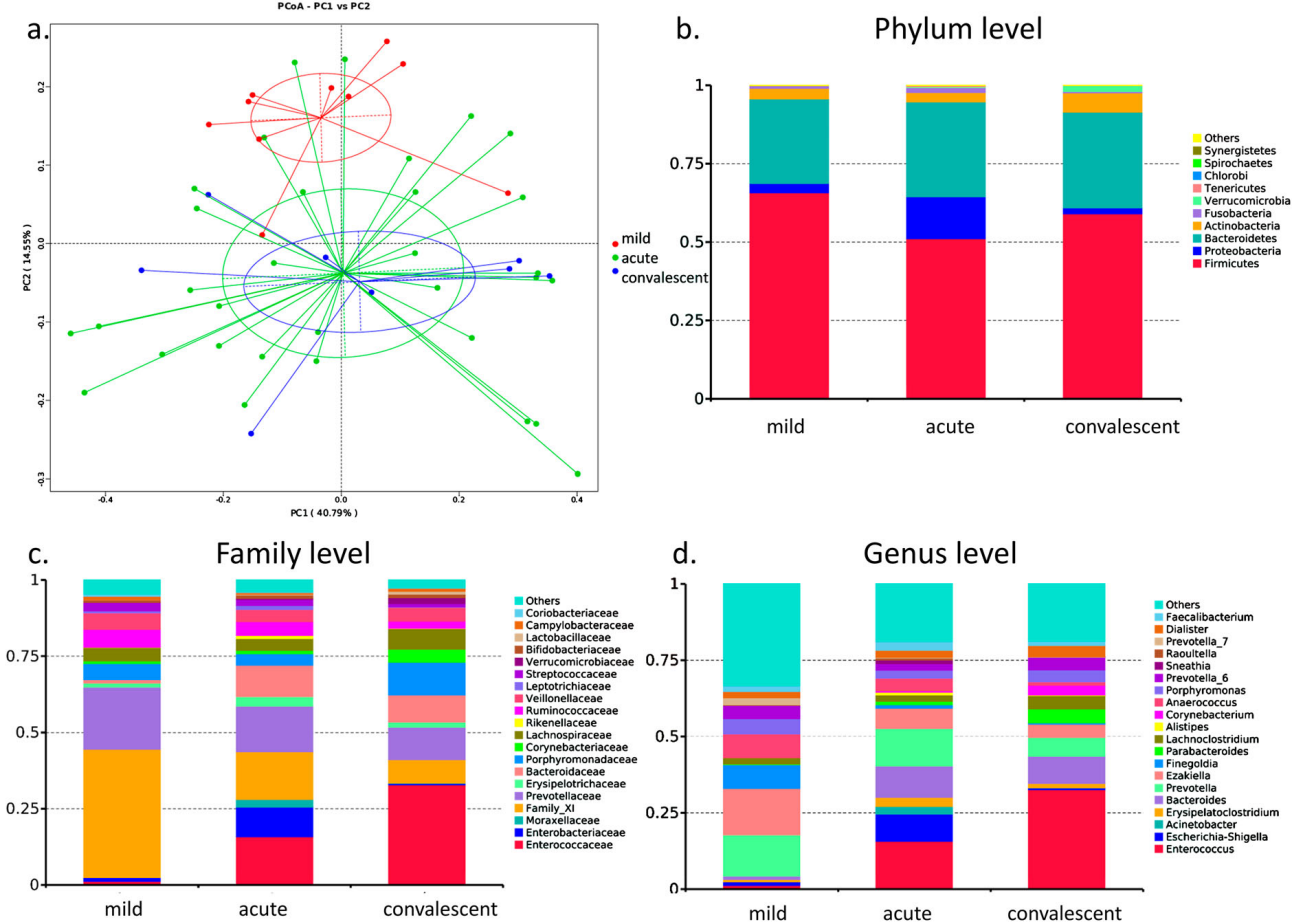
Bacterial composition of gut microbiota in patients with hand, foot, and mouth disease (HFMD). (a) Principal coordinate analysis of patients with mild and severe HFMD. For patients with severe HFMD, samples collected at acute and convalescent phases are included. Distribution of the predominant bacteria at phylum (b), family (c), and genus levels (d) are shown.

PCoA clearly showed a distinct separation in gut bacterial composition between patients with mild symptoms and patients with severe symptoms (Figure 1(a)). However, bacterial profiles were overlapped between the acute and convalescent phases in patients, suggesting that severe and mild cases had different bacterial profiles, whereas acute and convalescent phases exhibited similar bacterial profiles.

The gut microbiota of patients with mild HFMD was predominant in phylum Firmicutes (RA: 65.8%), its family Clostridiales Family XI (RA: 42.0%), and its genera *Ezakiella* (RA: 15.1%), *Peptoniphilus* (RA: 9.8%), *Finegoldia* (RA: 7.9%), and *Anaerococcus* (RA: 7.6%) (Figure 1(b,c)). These bacteria are considered as short-chain fatty acid (SCFA)-producing bacteria. The gut microbiota of patients at acute and convalescent phases showed similar composition. At the genus level, the gut microbiota at the acute phase in severe cases was predominant in *Enterococcus* (RA: 15.8%), *Prevotella* (RA: 12.3%), *Bacteroides* (RA: 10.3%), and *Escherichia-Shigella* (RA: 8.9%). Moreover, the gut microbiota in the convalescent phase in severe cases was predominant in *Enterococcus* (RA: 32.7%), *Bacteroides* (RA: 8.9%), *Prevotella* (RA: 6.2%), and *Parabacteroides* (RA: 4.6%).

LEfSe results showed that phylum Proteobacteria (RA: 3.0, 13.4 and 1.9%, *p* = 0.031), its family Enterobacteriaceae (RA: 1.3, 9.8 and 0.5%, *p* = 0.041, *p* = 0.027), its genus *Escherichia-Shigella* (RA: 1.2, 8.9 and 0.5%, *p* = 0.037), and its species *Escherichia coli* (RA: 1.2, 8.9 and 0.5%, *p* = 0.037) were enriched in the gut microbiota of severe cases at the acute phase (Figure 1(d)). Additionally, family Bacteroidaceae (RA: 1.1, 10.3, and 8.9%, *p* = 0.002), its genus *Bacteroides* (RA: 1.1, 10.3, and 8.9%, *p* = 0.002), and its species *Bacteroides fragilis* (RA: 0.5, 4.5 and 6.4%, *p* = 0.006) were also significantly increased in the gut microbiota of severe cases. Family Moraxellaceae and its genus *Acinetobacter* and family Erysipelotrichaceae and its genus *Erysipelatoclostridium* were also enriched in the gut microbiota of severe cases at the acute phase (*p* = 0.029, *p* = 0.021). Function prediction analysis also indicated that lipopolysaccharide (LPS) biosynthesis was markedly increased at acute phase.

LEfSe results also indicated that family Clostridiales Family XI (RA: 42.0, 15.7, and 7.7%, *p* = 0.001, *p *< 0.001) and its genus *Ezakiella* (RA: 15.1, 6.6, and 4.3%, *p* = 0.039), *Peptoniphilus* (RA: 9.8, 3.0 and 1.4%, *p* = 0.005, *p* = 0.001), *Anaerococcus* (RA: 7.6, 4.2 and 1.1%, *p* = 0.002, *p* = 0.011) and *Finegoldia* (RA: 7.9, 1.2, and 0.4%, *p* = 0.008), and species *Prevotella bivia* (RA: 5.9, 4.3, and 0.1%, *p* = 0.039) and *Prevotella corporis* (RA: 4.1, 0.6, and 0.1%, *p* = 0.031) were enriched in the gut microbiota of mild cases, whereas they were depleted in at the acute and convalescent phase in severe cases.

At the convalescent phase, family Enterococcaceae (RA: 1.2, 15.8, and 32.8%) and its genus *Enterococcus* (RA: 1.2, 15.8, and 32.7%) were enriched in the gut microbiota when compared with that at the acute phase and in mild cases (*p* = 0.007, *p* = 0.045). Meanwhile, RA of Firmicutes (RA: 59.0% and 65.8%), Bacteroidetes (RA: 27.0% and 30.2%), and Proteobacteria (RA: 1.9% and 2.9%) was restored to the levels observed in mild cases. Therefore, bacterial composition of the gut microbiota at the convalescent phase in severe cases was restored to some extent as that observed in mild cases.

### Severe cases exhibited significantly decreased diversity and richness of gut microbiota

**Figure 2.**
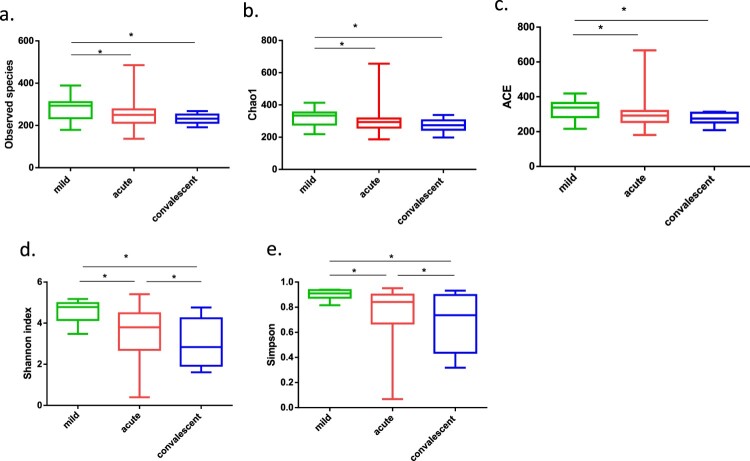
Alpha-diversity of gut microbiota in patients with mild and severe HFMD. For patients with severe HFMD, samples collected at the acute and convalescent phases are included. (a–e) Observed species, Chao 1, Abundance-based Coverage Estimator (ACE), Shannon index, and Simpson index, respectively. Statistically significant differences between the two groups are marked, and * indicates *p* < 0.05.

Compared to those in mild cases, all alpha diversity indices at the acute and convalescent phases of severe cases were significantly decreased (all *p*-values < 0.05). Specifically, the richness indices of observed species, Chao1, and ACE were decreased in the acute phase (*p* = 0.048, *p* = 0.044, and *p* = 0.037, respectively; Figure 2(a–c)). Moreover, the two evenness indices of Shannon and Simpson were significantly decreased in the convalescent phase compared with those observed in the acute phase (*p* = 0.033, *p* = 0.045, respectively; Figure 2(d,e)). This can be attributed to the marked and continuous decrease of Clostridiales Family XI and its genus *Ezakiella*, *Peptoniphilus, Anaerococcus,* and *Finegoldia*, and Prevotellaceae family and its genus *Prevotella*.

### Severe cases carried a predominant bacterial genus in gut microbiota

**Figure 3.**
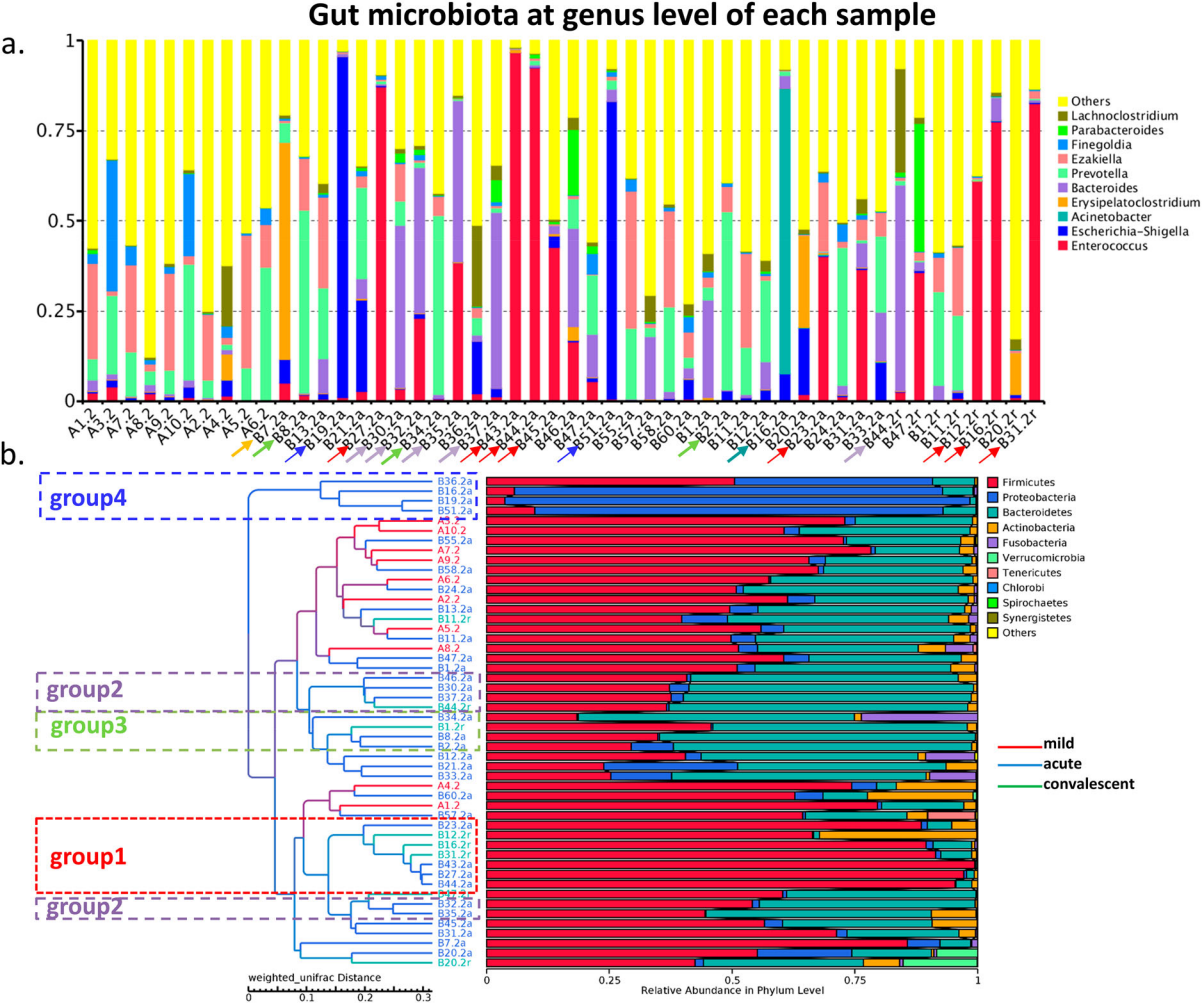
Gut bacterial composition of each sample. (a) Gut bacterial compositions of each sample at the genus level. (b) Tree based on weighted UniFrac distances of each sample at phylum level. Different coloured arrows represent different predominant bacteria, corresponding to different groups.

When one genus accounted for >40% of reads in a sample, it was defined as a predominant bacterial genus. On the basis of the top abundant genera, we found that 50% (*n* = 16) of severe cases harboured a predominant bacterium at the acute phase, including *Enterococcus* (*n* = 5, group 1), *Bacteroides* (*n* = 4, group 2), *Prevotella* (*n* = 3, group 3), *Escherichia-Shigella* (*n* = 2, group 4), *Erysipelatoclostridium* (*n* = 1), and *Acinetobacter* (*n* = 1) (Figure 3(a,b)). Meanwhile, 50% (*n* = 4) of severe cases carried a predominant bacterial genus at the convalescent phase, including *Enterococcus* (*n* = 3, group 1) and *Bacteroides* (*n* = 1, group 2). However, none of the mild cases carried any of these predominant bacteria. The number of cases with predominant bacteria was statistically different between the acute or convalescent phase and the mild cases (*p* = 0.001, *p* = 0.011).

### Gut bacterial profiles of patients infected with EV-A71, CV-A16, and other enteroviruses

Composition and richness of gut microbiota in patients with HFMD caused by EV-A71 and CV-A16 were analysed. Patients infected with EV-A71 had enriched Proteobacteria phylum, Enterobacteriaceae family, *Escherichia-Shigella* genus, and *E. coli* species (all LDA > 3; Supplementary Figure 1), whereas patients infected with CV-A16 had enriched Enterococcaceae and depleted Clostridiales Family XI (all LDA > 3). Patients infected by other enteroviruses exhibited bacterial profiles similar to those of patients infected with EV-A71. Additionally, function prediction analysis indicated that LPS biosynthesis was markedly enriched in patients infected with EV-A71.

### Oropharyngeal bacterial composition in patients with severe and mild HFMD

**Figure 4.**
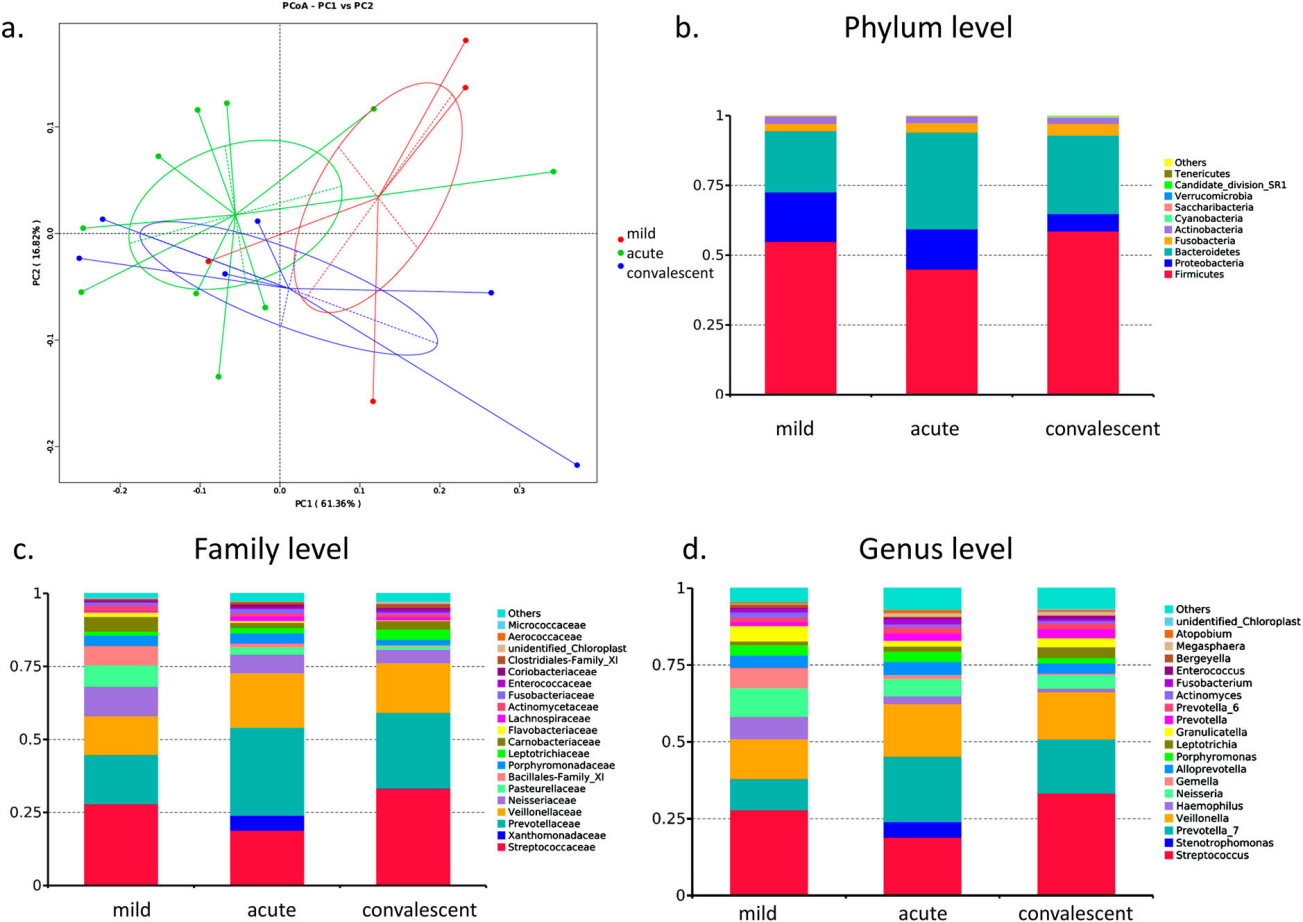
Bacterial composition of oropharyngeal microbiota in patients with hand, foot, and mouth disease (HFMD). (a) Principal coordinate analysis of patients with mild and severe HFMD. For patients with severe HFMD, samples collected at the acute and convalescent phases are included. Distribution of the predominant bacteria at the phylum (b), family (c), and genus levels (d) are shown.

PCoA did not distinguish the bacterial profiles in oropharyngeal microbiota between mild and severe cases, as well as between severe cases at the acute and convalescent phases (Figure 4(a)), suggesting that the three groups had a similar composition of oropharyngeal microbiota. Firmicutes, Proteobacteria, and Bacteroidetes, together accounting for 94.6%, 94.0%, and 93.0% of the sequences, respectively, were the top three most-abundant phyla (Figure 4(b)). Streptococcaceae and its genus *Streptococcus* (RA: 28.0, 19.0 and 33.4%), Prevotellaceae and its genus *Prevotella_7* (RA: 10.2, 21.3 and 17.5%), Veillonellaceae and its genus *Veillonella* (RA: 12.9, 17.1 and 15.5%), Neisseriaceae and its genus *Neisseria* (RA: 9.3, 5.8 and 4.4%), and Pasteurellaceae and its genus *Haemophilus* (RA: 7.2, 2.5 and 1.1%) were the dominant families and genus in the three groups (Figure 4(c,d)). Altogether, *Streptococcus*, *Prevotella,* and *Veillonella* accounted for 51.1%, 57.4%, and 66.4% of the sequences in the three groups, respectively.

LEfSe results showed that Bacilli, Bacillales, Family_XI, *Streptococcus*, and *Gemella* were significantly enriched in the oropharyngeal microbiota of mild cases compared with that in severe cases in the acute and convalescent phases (all LDA > 3). In the acute phase, Clostridia (*p* = 0.009), Clostridiales (*p* = 0.009), Megasphaera (*p* = 0.038), Lachnospiraceae (*p* = 0.026), Ruminococcaceae (*p* = 0.024), and *Alloprevotella* (*p* = 0.028), and their species were significantly increased compared with the mild cases. Convalescent phase showed significant enrichment of Clostridia (*p* = 0.018), Clostridiales (*p* = 0.018), and *Streptococcus_salivarius* (*p* = 0.026) compared with the mild cases. There was no significant difference in diversity and richness between the three groups. Patient B16 in acute phase was enriched in Xanthomonadaceae family, its genus *Stenotrophomonas* (RA: 5.1%), and its species *Pseudomonas geniculate* (RA: 50.5%).

### Predominant bacterial genus observed in the oropharyngeal microbiota of patients with severe HFMD

**Figure 5.**
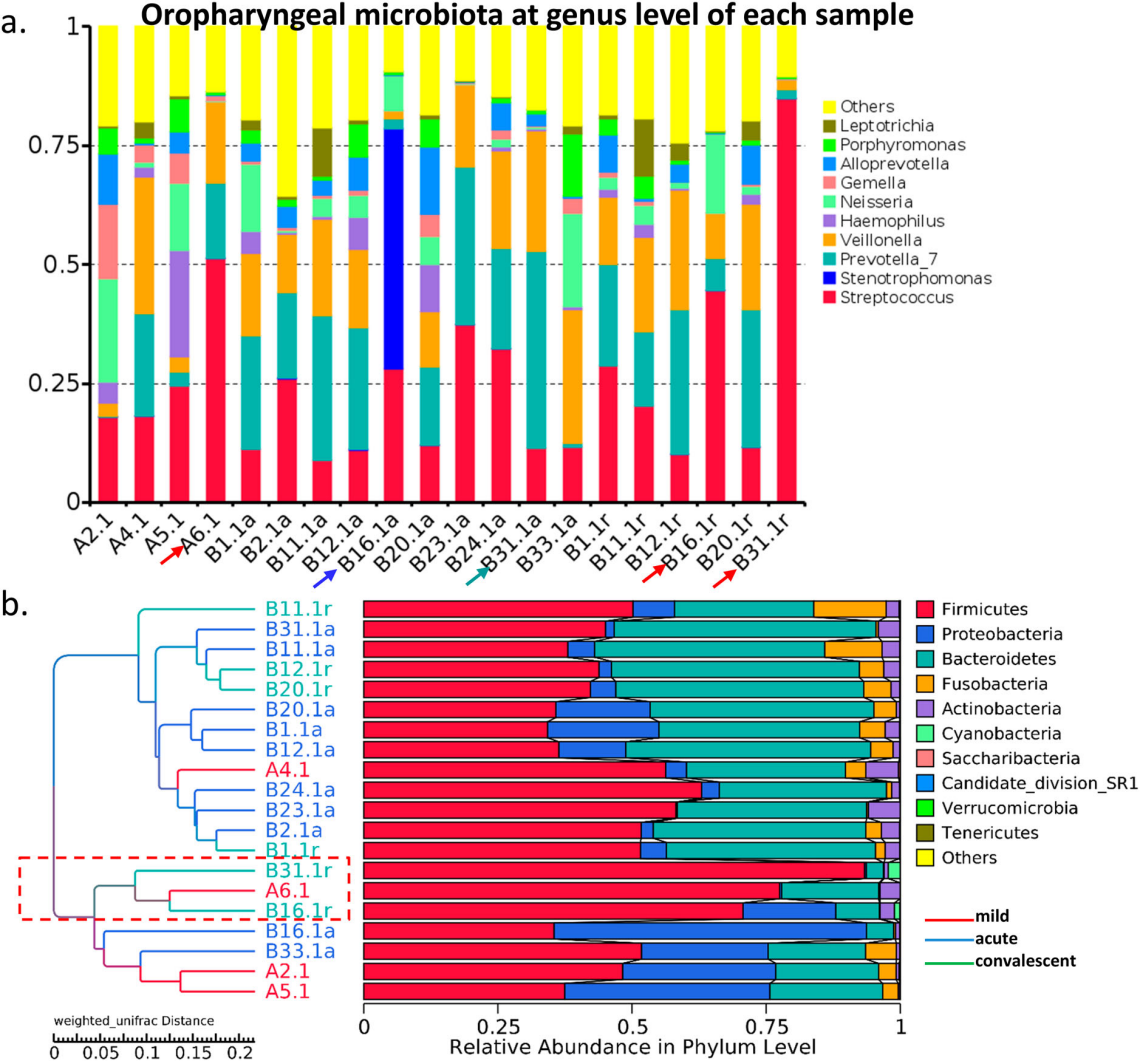
Oropharyngeal bacterial composition of each sample. (a) Bacterial composition of each sample at the genus level. (b) Tree based on weighted UniFrac distances of each sample at phylum level. Different coloured arrows represent different predominant bacteria, corresponding to different groups.

Six patients (B1, B11, B12, B16, B20, and B31) were provided oropharyngeal swabs for acute and convalescent phases (Table 1). Of them, two patients at acute phase (B16 and B31) were predominant in *Stenotrophomonas* and *Prevotella_7*, respectively. Two patients at convalescent phase were predominant in *Streptococcus* (Figure 5(a,b)). Notably, gut microbiota in the two patients were also predominant in *Enterococcus* and *Acinetobacter* at the acute phase and all *Enterococcus* in the convalescent phase, respectively.

### Correlation between gut and oropharyngeal microbiota

**Figure 6.**
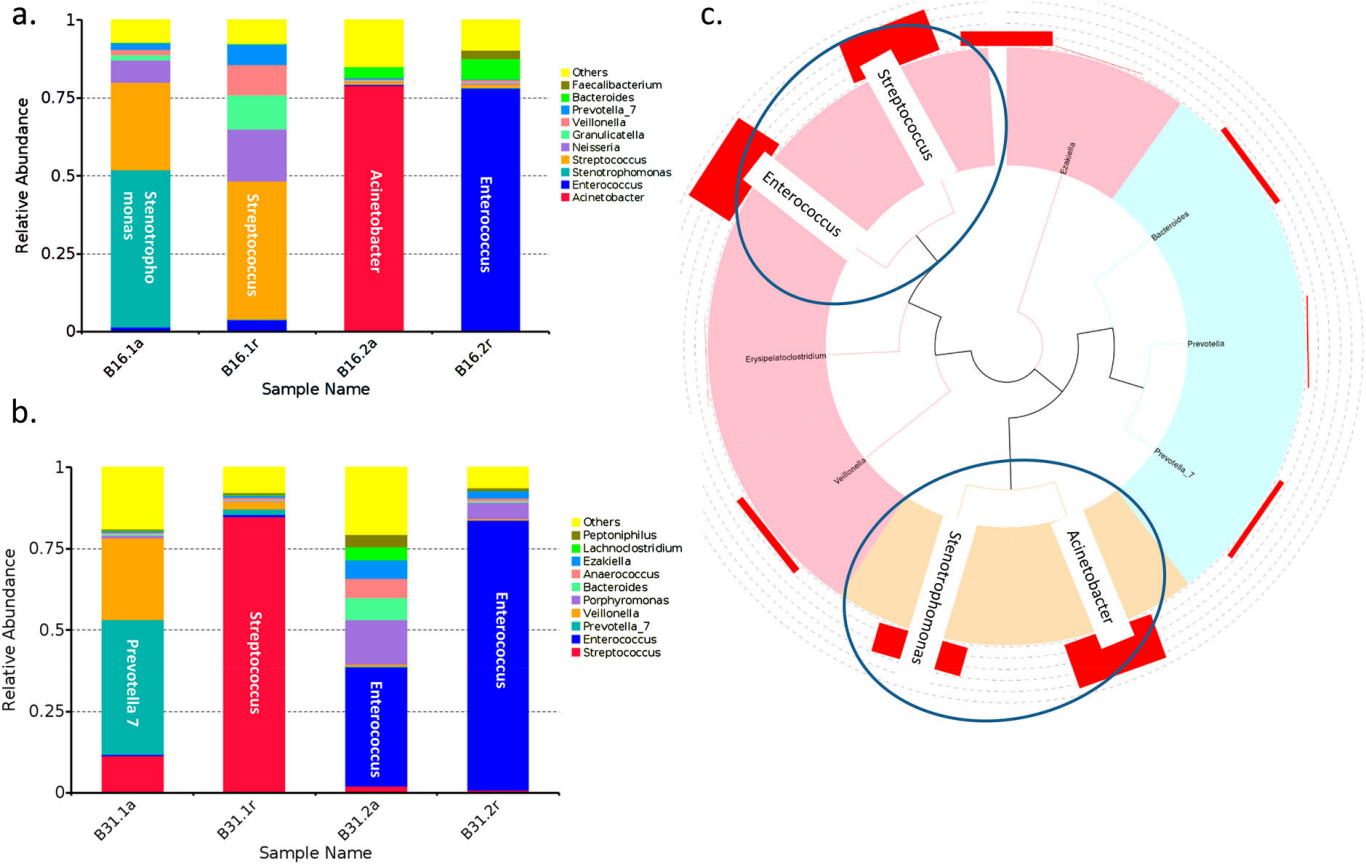
Example of the predominant bacteria carried by patients B16 and B31. (a) The predominant bacteria carried by patients B16 and B31 in the gut and oropharynx at acute and convalescent phases. (b) Phylogenetic tree of the predominant bacteria carried by the two patients.

Two serial rectal and oropharyngeal swabs at acute and convalescent phases, respectively, were collected from six patients during their hospitalization. We thus studied the correlation between predominant bacteria in the gut and oropharyngeal microbiota by analysing the paired-collected samples. We found predominant bacteria in gut and oropharynx at acute and convalescent phases in two patients (B16 and B31). In patient B16, *Stenotrophomonas* and *Acinetobacter* were predominant in the oropharynx and the gut, respectively, at acute phase (Figure 6(a)). While oropharyngeal microbiota of B16 and B31 was predominant in *Streptococcus*, and *Enterococcus* was predominant in the gut microbiota at the convalescent phase (Figure 6(b)). Phylogenetic analysis showed that genus *Stenotrophomonas* and *Acinetobacter* and *Enterococcus* and *Streptococcus* were derived from the same common origin, respectively (Figure 6(c)).

## Discussion

We found that severe cases exhibited depleted SCFA-producing bacteria and were more frequently predominant in inflammation-inducing bacteria. These bacteria included *E. coli*, *B. fragilis*, *Enterococcus*, *Prevotella*, *Erysipelatoclostridium,* and *Acinetobacter* in the gut, and *Streptococcus*, *Prevotella,* and *Stenotrophomonas* in the oropharynx. We also successfully mapped out both the gut and oropharynx microbiota of pediatric patients with HFMD in China. To the best of our knowledge, this study is the first to show dynamic changes in bacterial composition of the gut and oropharyngeal microbiota among patients with HFMD.

An epidemiological study reported that few or no mild cases developed into severe cases, whereas severe cases usually showed quick progression, resulting in death in some instances within 1–3 days after the appearance of symptoms [3]. This suggests that mild and severe cases of HFMD might be independent of each other and have different causes and underlying mechanisms. Our results partially confirmed this result. Our findings demonstrated a significant shift in gut microbiome composition in patients with severe HFMD, especially at the acute phase. The dysbiosis observed was characterized by the overgrowth of pathogenic and inflammation-inducing bacteria and depletion of butyrate-producing bacteria in comparison with mild HFMD. These characteristic changes were also observed in patients with HFMD when compared with healthy individuals [9]. The overgrowth of Enterobacteriaceae members might lead to the release of large amounts of LPS. Increased amounts of LPS have been linked to low-grade mucosal inflammation [32]. This is mainly due to the disruption of intestinal barrier integrity and the increase of its permeability, which facilitates microbial translocation [17,32]. Previous data have confirmed that EV-A71 infects and replicates in intestinal epithelial cells by activating p38 mitogen-activated protein kinase and protein kinase signalling pathways [33]. This inflammatory pathway overlaps with the inflammatory reactions caused by bacterial LPS or translocation. This is consistent with our results, wherein patients infected with EV-A71 were enriched in Enterobacteriaceae family. *Enterococcus* has also been implicated in impaired intestinal permeability and facilitated microbial translocation [17]. In particular, it can induce a higher susceptibility to intestinal inflammation by producing gelatinase, which is a metalloprotease that impairs the epithelial barrier. Enterotoxigenic *B. fragilis* (ETBF) can produce enterotoxins and induce the release of pro-inflammatory cytokines, such as interleukin (IL)-6, tumour necrosis factor (TNF)-α, and CXC motivating ligand (CXCL)-8, by intestinal epithelial cells [34]. Additionally, ETBF can increase the level of Th17 lymphocytes and enhance STAT-3-dependent Th17 immune response [35]. *B. fragilis* and *Enterococcus faecalis* have been shown to produce reactive oxygen species, which drive oxidative damage of DNA and inflammation in epithelial cells of the gut [36]. Subsequently, microbial products, including LPS and polysaccharide A, along with induced cytokines make their way across the damaged barrier into the blood circulation of the host, thereby causing systemic inflammation [37,38]. This is one answer to why patients with severe HFMD usually display a “cytokine storm.” Indeed, many patients with severe HFMD exhibited a significant increase in inflammation-causing bacteria in our study.

The number of species and diversity of oropharyngeal and gut microbiota have been shown to increase dramatically in the first year of life and become stable afterwards [39]. In healthy Chinese children aged 2 years old, the dominant genera in the oropharynx include *Streptococcus* (RA: 28.5–31.8%), *Prevotella* (RA: 16.1–17.3%), *Neisseria* (RA: 12.6–13.1%), *Veillonella* (RA: 8.1–9.0%), and *Haemophilus* (RA: 6.2–6.3%) [20,21]. In our study, both mild and severe cases exhibited similar composition of oropharynx microbiota. However, differences in RA of these genera were found among patients with severe HFMD at both the acute and convalescent phases in comparison to healthy children. For example, opportunistic pathogen *Veillonella* were increased (17.1, 15.5 vs. 9.0%), and *Neisseria* (5.8, 4.4 vs. 12.6%) and *Haemophilus* (2.5, 1.1 vs. 6.2%) were decreased. However, these differences were not significant. In contrast, patients with mild HFMD did not exhibit such changes. In a recent study, Ma et al. compared oropharyngeal microbiota between patients with COVID-19 and healthy controls and found significant enrichment of opportunistic pathogens including *Veillonella* genus. They concluded that oropharyngeal microbiota alterations and functional differences were associated with COVID-19 severity [40]. Our result was in agreement with the finding of the above study. Patients with HFMD had different gut bacterial composition compared with healthy controls regardless of disease severity. The gut microbiota of healthy children was predominant in *Bacteroides*, *Faecalibacterium*, *Bifidobacterium*, *Prevotella, Fusobacterium, Pseudobutyrivibrio,* and *Lachnoclostridium* [22,23], whereas the gut microbiota in mild cases was predominant in butyrate-producing bacteria including *Ezakiella, Prevotella, Peptoniphilus, Finegoldia*, and *Anaerococcus*. The gut microbiota in severe cases was dominant in inflammation-inducing bacteria including *Enterococcus, Prevotella, Bacteroides,* and *Escherichia-Shigella.* The clinical and immunological relevance of the changes in genera common to both the oropharynx and gut and how long these changes will last remains to be elucidated.

Our results also showed that the oropharyngeal microbiota in patients with severe HFMD was predominant in several bacterial genera, including *Stenotrophomonas*, *Prevotella,* and *Streptococcus*. The results are in line with several recent studies on oropharyngeal microbiota of patients with COVID-19 [41,42]. These bacteria were also observed in influenza virus-infected patients who often had secondary bacterial pneumonia. SDPG in the oropharyngeal microbiota has been shown to be associated with a secondary bacterial infection in influenza virus-infected patients [18]. More than 60% of patients with severe influenza carried SDPG with predominant *Streptococcus*, *Prevotella*, and other related genus. These bacterial species belonging to the SDPG can subsequently cause severe pneumonia and bacteraemia with high mortality. Another recent study reported that the proportion of patients with severe COVID-19 with SDPGs in their airways was significantly higher than that of patients with mild COVID-19 [19]. *Streptococcus* is one of the most prevalent inhabitants of the oropharynx and respiratory tract of adults and children. Recent study has reported the reduction in proportion of *Streptococcus* and *Velionella* in COVID-19-infected patients compared to healthy control [41]. *Prevotella* members exhibit pro-inflammatory properties. Previous studies have shown that its relative abundance in the oral microbiota or in the nasopharyngeal microbiota was positively associated with long COVID or COVID-19 severity, respectively [43,44]. Moreover, Lai et al. have also reported that *Prevotella*_7 in the oropharyngeal microbiota were significantly enriched in patients with COVID-19 and positively correlated with the level of the inflammation biomarker C-reactive protein [42]. *Stenotrophomonas* is known as an organism with a low number of virulence factors, including efflux drug pumps, flagella, and siderophores, which cause opportunistic infections. Recently, Paine et al. reported that it was significantly associated with COVID-19 infection [41]. Therefore, it remains to be studied whether these enriched bacterial genera could contribute to the progression and severity of HFMD.

Our study has several limitations. First, the number of participants was limited. Upon dividing patients into groups of mild and severe symptoms, the sample size of each group became even smaller. When patients with HFMD caused by EV-A71 and CV-A16 were compared, the number of patients caused by CV-A16 was only 3. There seemed difference in gut microbiota composition and richness between two groups. This result was consistent with the previous study [8]. Further studies with a large number of participants are needed. Second, since rectal swabs are more convenient to be collected from children and pediatric patients, we used rectal swabs rather than stool samples for gut microbiome analysis. Studies, including our own, have shown that both OTUs numbers and bacterial composition identified in rectal swabs were rather similar to those in stool for children [45–47]. Third, the samples at convalescent phase were mainly collected 7–12 days after the onset of the disease. Samples for convalescent phase were collected on the same day when patients were discharged from the hospital. Although it is possible for some patients who did not fully represent the time of microbial restoring, bacterial compositions of convalescent phase of severe patients restored, to some extent to those observed in mild patients. Fourth, this study did not include healthy children as controls. This study was designed to investigate and compare microbiome dysbiosis between mild and severe patients with HFMD.

In conclusion, this study demonstrated that diversity of gut microbiota was significantly impaired and inflammation-inducing bacteria were frequently detected in severe patients with HFMD. A shift in gut microbiota composition was found from acute to convalescent phase of HFMD in the patients. In addition, the predominant bacteria in oropharynx carried by severe patients seemed to resemble those observed in their gut. Further studies with a large number of participants should be conducted to validate the findings of our study.

## Supplementary Material

Supplemental Material
