# Supplementary figures and images for: Integrated analysis reveals important differences in the gut and oropharyngeal microbiota between children with mild and severe hand, foot, and mouth disease
Source: Emerg Microbes Infect. 2023 Mar 31;12(1):2192819. doi: 10.1080/22221751.2023.2192819 (PMC10071984; doi:10.1080/22221751.2023.2192819)

## Slide 1
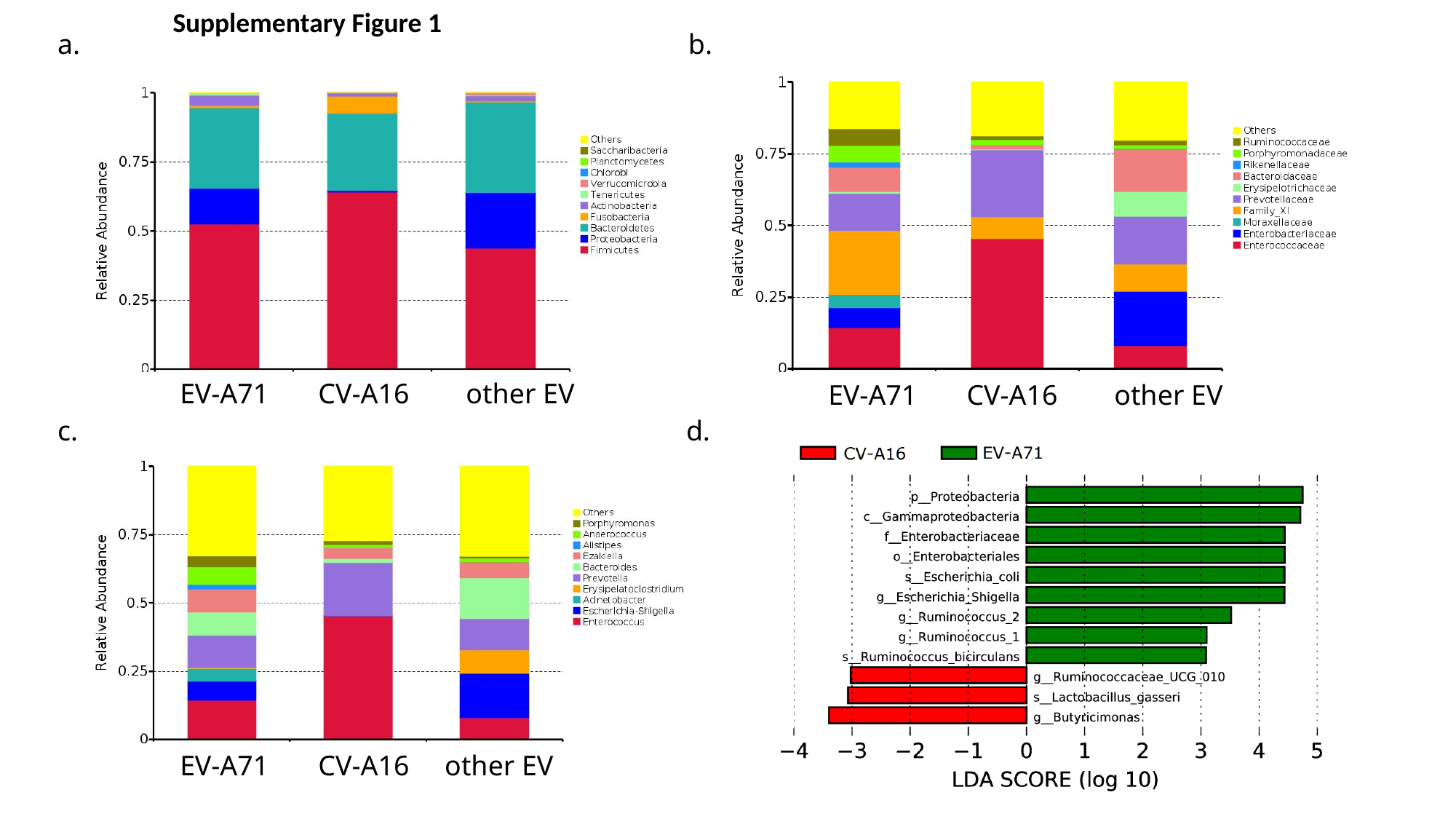

Supplementary Figure 1
a. b.
EV-A71 CV-A16 other EV
EV-A71 CV-A16 other EV
c. d.
EV-A71 CV-A16 other EV

Supplement: Supplemental Material [file TEMI_A_2192819_SM8915.pptx]
